# The relationship between empathy and altruistic motivations in nursing studies: a multi-method study

**DOI:** 10.1186/s12912-021-00620-4

**Published:** 2021-07-07

**Authors:** Linda Messineo, Luciano Seta, Mario Allegra

**Affiliations:** Istituto per le Tecnologie Didattiche, Consiglio Nazionale delle Ricerche, via Ugo La Malfa 153, 90146 Palermo, Italy

**Keywords:** Degree choice, Empathy, Jefferson scale of empathy – health professions students version, Thematic analysis, Undergraduate students

## Abstract

**Background:**

The efficient management of relational competences in healthcare professionals is crucial to ensuring that a patient’s treatment and care process is conducted positively. Empathy is a major component of the relational skills expected of health professionals. Knowledge of undergraduate healthcare students’ empathic abilities is important for educators in designing specific and efficient educational programmes aimed at supporting or enhancing such competences. In this study, we measured first-year undergraduate nursing students’ attitudes towards professional empathy in clinical encounters. The students’ motivations for entering nursing education were also evaluated. This study takes a multi-method approach based on the use of qualitative and quantitative tools to examine the association between students’ positive attitudes towards the value of empathy in health professionals and their prosocial and altruistic motivations in choosing to engage in nursing studies.

**Methods:**

A multi-method study was performed with 77 first-year nursing students. The Jefferson Scale of Empathy (JSE) – Health Professions Student Version was administered. Students’ motivations for choosing nursing studies were detected through an open question and thematically analysed. Using explorative factor analysis and principal component analysis, a dimensional reduction was conducted to identify subjects with prosocial and altruistic motivations. Finally, linear models were tested to examine specific associations between motivation and empathy.

**Results:**

Seven distinct themes distinguishing internal and external motivational factors were identified through a thematic analysis of students’ answers regarding their decision to enter a nursing degree course. Female students gained higher scores on the empathy scale than male ones. When students’ age was considered, this difference was only observed for younger students, with young females’ total scores being higher than young males'. High empathy scores were positively associated with altruistic motivational factors. A negative correlation was found between external motivational factors and the scores of the Compassionate Care subscale of the JSE.

**Conclusions:**

Knowing the level of nursing students’ empathy and their motivational factors for entering nursing studies is important for educators to implement training paths that enhance students’ relational attitudes and skills and promote the positive motivational aspects that are central to this profession.

## Background

In recent decades, there has been a growing amount of empirical scientific research on the importance of positive relationships between health professionals and patients to ensure improvement of the cure and treatment process [1]. Health specialists need to master interpersonal and communication skills, including willingness to listen, grasping what patients communicate with interest, and being aware of patients’ attitudes and psychological characteristics. Several studies have attempted to determine whether interactions between clinicians and patients have a beneficial effect on health-related outcomes. Kelley et al. [1] conducted a systematic review and meta-analysis of research in which this specific relationship was examined. They suggested that the clinician-patient relationship has a small but statistically significant effect on healthcare outcomes such as blood pressure reduction or pain scores. Healthcare professionals with good interpersonal skills and empathy towards patients have a positive and significant impact on patient satisfaction, adherence to treatment and care outcomes [1]. Empathy is a meaningful factor in the healthcare professional-patient relationship. Moreover, it is one of the most powerful personal attributes that health professionals can use to encourage patients to modify their health, producing positive clinical outcomes [1, 2]. Patients should perceive health professionals as empathic people. Empathy allows patients to feel understood, validated, and respected. Studies have identified empathy as a useful skill for nurses regarding its impact on the improvement of patient outcomes such as distress and anxiety reduction [3]. Much attention has been dedicated to this issue, and the importance of healthcare personnel taking an empathic approach has been stressed [4]. Further, many educational programmes have focused on promoting empathy in undergraduate students and healthcare professionals [5–7]. However, debates among researchers regarding the description and operationalisation of empathy are still open, with the related considerations about specific instruments for empathy measurement in the health sector, in general, and in nursing, in particular [4, 8–11]. Empathy does not have a clear and unambiguous definition [8]; it is a multi-dimensional construct characterised by cognitive and affective aspects [4]. Cognitive empathy refers to the ability by which health professionals understand patients’ experiences and emotions and have the competence to communicate this understanding to patients. Emotional empathy is related to the emotional responses of participation of healthcare personnel in patients’ feelings. In a caring relationship, such as the nurse-patient relationship, empathy is principally characterised by the cognitive component [4]. This competence is developed over the course of one’s life; all individuals are typically found to have intermediate levels of this ability, though they fall at different locations on the empathy spectrum. Research has shown that health profession students’ empathy declines over the course of their training [12–14]. Conversely, other studies have reported no change or an increase of empathy during undergraduate education [15–17]. Promoting empathy is one of the objectives of healthcare education [5–7]. It is important for educators to understand undergraduate healthcare students’ empathy levels in order to tailor educational programmes to support or enhance students’ empathic competences. Various instruments have been created to measure empathy levels in healthcare-related contexts [8, 9, 11]. One of the most commonly used surveys to measure empathy in the healthcare sector is the Jefferson Scale of Empathy (JSE) [18]. Different versions of the JSE have been developed for physicians and other health professionals (HP version), for medical students (S version), and for students of other healthcare specialties (HPS version) [4], the last of which was used in this study.

Prior research has focused on the association between medical students’ empathy and their motivation for studying, an important factor which may influence academic achievement and student retention [19]. A weak association between empathy and reasons for enrolling in medical courses has been observed by some researchers [20]. Another study showed a significant association between the empathy scores of first-year medical students and their intention to pursue people-oriented specialties after graduation [21]. A recent study showed a significant positive association between internal motivational factors (such as altruism or caring for patients) for studying medicine and empathy [22]. Another study concluded that there is no association between the JSE scores and speciality interest in osteopathic medical students [23]. No studies have investigated the association between students’ positive attitudes towards the value of empathy in health professionals and their motivation for engaging in nursing studies. Students’ motivations and reasons for choosing to take a nursing university course and follow nursing as a career have been explored in different studies. Such research has found that these choices are the result of a combination of internal and external motivational factors; sometimes, nursing is not students’ first options. Students choose to engage in the nursing profession based on their desire to help other people and engage in activities and perform work with social benefits [24–27]. External motivational factors, such as career opportunities and job security, are also important in students’ career choices [24, 26, 27]. Personal health-related experiences such as hospitalisation, the illness of a family member, or a volunteering experience, have been detected as additional motivations [25, 27]. Furthermore, some students choose nursing studies due to their interest in scientific subjects. Personal experiences and family members and friends can play key roles as main sources of information for nursing students concerning the nursing profession [28, 29]. Internal motivational factors for entering nursing, such as helping others, are the motivational factors most commonly indicated by students.

In the present study, we measured empathy of first-year undergraduate nursing students and their motivations to pursue nursing education. We expected to find an association between students’ positive attitudes towards the value of empathy in health professionals and their altruistic motivations for choosing nursing studies. Altruistic motivations refer to the desire to perform voluntary actions in order to generate a benefit for others, promote well-being, and alleviate others’ needs [30].

### Research hypotheses

The primary aim of this study is to describe the association between empathy and reasons for enrolling in nursing. We expected to find a significant association between empathy scores and altruistic motivations for choosing nursing studies.

The secondary aim of this study is to verify whether there are significant age and sex differences in empathy scores and nursing motivations. According to the existing literature, we expected to find significant sex differences in empathy scores [20, 22, 31]. We also expected to identify age and sex differences with respect to nursing motivations [27].

## Methods

### Study design

Considering the explorative nature of this study, which was aimed at determining the existence of a relationship between empathy and motivation, a multi-method research approach was adopted. A quantitative tool was used to measure empathy; answers to an open question were thematically analysed to evaluate nursing motivation.

### Setting and data collection

All students (*N* = 120) enrolled in the first year of the nursing degree course at the Medical School of a university in southern Italy were invited to participate in the study. Data were collected at the beginning of the first academic semester. Students were informed about the aim of the research and the study procedure just after class time by one of the researchers (LM), a general psychology aggregate professor in the nursing degree course. To reduce any perceived pressure to participate in the study, as their professor was also the researcher, students were informed that they were free to choose whether to participate or not, with no negative consequence for their study career. They were also informed that all data collected were anonymous and the professor was unaware of which students participated and which did not. After receiving all information, the students filled in the online questionnaire structured for the purposes of the present study.

### Ethics

According to the local ethical policy, no formal approval by the ethics committee was necessary. We communicated the study design to the institutional board of the degree course, guaranteeing that ethical standards would be met, and received consent. The confidentiality of the collected data was guaranteed, and the professor was unable to match students and responses. Participation was voluntary, and students could decide to withdraw their participation at any moment without any consequence. Before answering the questionnaire, participants provided written informed consent.

### Instruments and measures

The students completed a three-part questionnaire to measure socio-demographic factors, their motivation for choosing nursing studies, and their empathy levels.

#### Socio-demographic factors

Through a few questions, some socio-demographic aspects were collected, such as sex and age.

#### Motivation for choosing nursing studies

An open question was administered to students to gather information regarding their motivations for choosing to enrol in a university nursing course. Students were asked to answer the following question: *Describe your motivations for choosing a degree course in nursing.* This open question stimulated students to think independently instead of choosing from predetermined responses in a structured questionnaire.

#### Attitudes towards empathy

Permission to use the Italian adaptation of the Health Professions Students version of the JSE (JSE-HPS) was obtained to measure students’ orientation towards the value of empathy in health professionals in clinical contexts [4, 32]. The JSE-HPS is a self-report instrument and includes 20 items answered on a 7-point Likert scale (1 = strongly disagree, to 7 = strongly agree). The questionnaire comprises three factors: a) perspective taking, b) compassionate care/emotional engagement, and c) standing in the patient’s shoes. The survey was developed based on a robust research literature review regarding empathy activated in a relationship aimed to treat and cure a patient, in which health professionals acquire cognitive comprehension about patients’ concerns and their general vision of health and illness, and have abilities to communicate this understanding to patients. A higher score on the JSE-HPS scale (the score range is 20 to 140) reflects a greater inclination towards empathic involvement in patient treatment. Studies have showed that females generally have significantly higher JSE scores than males [20, 22, 31].

### Qualitative data analysis

The students’ answers to the open question were analysed using a thematic analysis to investigate their motivations for engaging in nursing studies [33]. Two independent evaluators (LM and LS) followed six phases to categorise the students’ responses. During each phase of the thematic analysis, specific activities were performed to establish trustworthiness [34]. In the first phase, the two researchers read and familiarised themselves with the qualitative data, which consisted of 77 responses. They stored the data in well-organised archives and considered potential themes. After an in-depth reading of the responses, during the second phase, the two researchers independently conducted an analytic segmentation of the contents, with the aim of generating initial codes and recognising themes. The credibility of the thematic analysis was enhanced by engaging two evaluators in an analysis of the qualitative data. Periodic meetings were held throughout the coding procedure for peer debriefing. During phase three, the researchers identified different analytical units in each section of the texts, such as words, phrases, statements, or entire paragraphs, using similar words or expressing the same ideas, from which to extract meaningful core themes. They kept detailed notes about the development of themes. This process of inferential analysis, performed independently by the two evaluators, was followed by a discussion aimed at selecting relevant categories. Afterwards, during phase four, a second examination of the students’ answers was conducted to refine the correspondence between content and selected categories. During phase five, themes were revised and defined, with the description of a set of codes representing single words or short phrases that best described the motivations for the students’ decisions. A meeting was held to discuss each of the revealed themes, and the few differences were resolved through discussion. The result was produced during phase six and consisted of the creation of a report describing the process of coding and analysis, with the inclusion of representative quotes for each theme and a set of codes representing single words or short phrases that best described the motivations for the students’ decisions.

### Bias control

To avoid selection bias, the sample was selected at the start of the academic year. To avoid information bias, the questions and respondents’ answers were presented and gathered in written form. To avoid interpretation bias of the selected information, the following precautions were taken: a) evaluation of responses to the open question was carried out inductively and not using a priori categories, by two different researchers; b) the analysis was conducted following the principal theoretical approaches to the motivation; and c) the findings of the thematic analysis were compared with the categories described in the relevant literature.

### Quantitative data analysis

Data were analyzed using the R and Statistical Package for Social Sciences (SPSS). The quantitative approach was applied to analyse the discrete variables, the JSE-HPS subscale and total scores, obtained by summing the respective item scores of the JSE-HPS. Simple non-parametric tests, namely the Kruskal-Wallis and Mann-Whitney tests, were applied to examine the existence of sex or age differences in the scores of the sample population. Moreover, the frequency distribution of the occurrences of the motivational categories in the sample was analysed to detect possible significant differences regarding sex and age.

To group the categories according to the patterns of co-occurrence of the motivational categories in the students’ answers, a first hierarchical cluster analysis was conducted, using the R function ‘heatmap’, where the heat scale corresponded to the correlation matrix values. The results of this analysis were confirmed with the SPSS software.

In a second step, a person-centred analysis was conducted to better understand the structure of the motivations that emerged from the thematic analysis. A person-centred analysis is aimed at clustering students according to a specific set of variables. In this study, this analysis was based on the application of the principal component analysis (PCA).

The properties of the correlation matrix, related to the limited sample size and the relative sporadic occurrence of the various categories, only partially supported the possibility of conducting the PCA. From the explorative factor analysis, we observed three components with eigenvalues larger than 1, explaining 63% of the total variance (KMO test < .50, Bartlett test: *χ* ^*2*^ = 23.67, *df* = 15; *p* < 0.1). The good agreement between the results of these two different methods corroborated the validity of the results of this study.

The PCA made it possible to attribute three motivational scores to each student, so that three continuous variables were associated to each student for each principal component, according to the pattern of themes identified in the student’ answer to the open question. Using the students’ motivational scores, linear models were tested to study the associations between specific motivational components and empathy scores.

## Results

### Demographic characteristics

Of the 120 first-year nursing students, 77 (64.17%) completed all three parts of the questionnaire. Respondents’ mean age was 21.53 (*SD* = 4.02). The sample comprised 37.7% males (*n* = 29; *M* = 23, *SD* = 3.36) and 62.3% females (*n* = 48; *M* = 20.48, *SD* = 4.80).

### Motivation for nursing studies

The thematic analysis of students’ answers to the open question concerning their choice of a nursing degree course identified seven distinct themes: a) willingness to care for people; b) human contact; c) healthcare-related personal experiences; d) personal interest in scientific topics; e) job opportunities; f) family tradition; g) other.

On average, two categories were selected for each subject (*M* = 1.86, *SD* = 0.94). Table 1 summarises the identified themes and subthemes of the thematic analysis, with a brief description of each. The category ‘Other’ was not included in the following analyses due to their scarcity and heterogeneity.

**Table 1:** Inductively inferred categories

Theme	Brief description	Illustrative quotes
HEL	Willingness to feel useful and to affect the health of others.	*‘I want to be of help to others; I want to contribute to trying to improve the lives of others.’* *‘I chose this degree programme to make myself useful for others’*
HUM	The desire to enter into contact with other people.	*‘I chose the degree course in nursing ... because I like being in contact with people.’* *‘I love being in contact with people.’*
FAM	Significant others, such as family members, have significant influence on the students’ career decision.	*‘Also, because there is a nurse in my family, that is, my grandmother, who was always an example for me.’* *‘When my brother, also a nurse, told me about his experiences in the ward, I was fascinated.’*
TOP	A personal interest in health-related scientific topics.	*‘I chose this course of study because I like the subjects in this degree course.’* *‘First of all, because I love the subjects that are studied in the nursing degree course.’*
JOB	The possibility of secure employment and job stability.	*‘I chose the undergraduate degree in nursing... to have greater job opportunities after finishing my studies.’* *‘Another reason … is that I hope to find work faster.’*
EXP	Healthcare-related experience, such as voluntary work in healthcare settings or a family member’s hospitalisation.	*‘I have chosen to undertake these studies and subsequently enrol in the degree course in nursing because I feel alive only when I help and support others and I received confirmation following the illness of a person to whom I was very attached.’* *‘I found myself at home in close contact with a grandfather to look after, I was his personal nurse, and this made me a happy person.’*
Other	Not being able to get into other chosen study programmes, etc.	*‘…I tried (to get into) the medicine degree course, but I wasn’t admitted.’*

As shown in Table 2, the highest reported motivation was the willingness to care for and help others (HEL). The second most frequently stated motivation concerned job opportunities (JOB). The category related to social influences (FAM) rarely appeared in students’ reported answers.

**Table 2:** Absolute and relative frequencies of motivation categories; relative frequencies of categories with respect to students’ sex and ages

	n	Tot (%)	Females (%)	Males (%)	Young (%)	Mid (%)	Old (%)
EXP	14	18.18	25.00	7.41	18.75	22.22	11.11
HUM	19	24.68	27.08	22.22	25.00	29.63	16.67
HEL	57	74.03	83.33	62.96	75.00	81.48	61.11
FAM	5	6.49	4.17	11.11	3.12	7.41	11.11
JOB	22	28.57	18.75	48.15	28.12	22.22	38.89
TOP	17	22.08	16.67	33.33	18.75	25.93	22.22

Young: students with age ≤ 19 years; Mid: students with age > 19 and ≤ 21 years; Old: students with age > 21 years

*EXP* Healthcare-related personal experiences, *HUM* Human contact, *HEL* Willingness to care for people, *FAM* Family tradition, *JOB* Job opportunities, *TOP* Personal interest in scientific topics

**Fig. 1:**
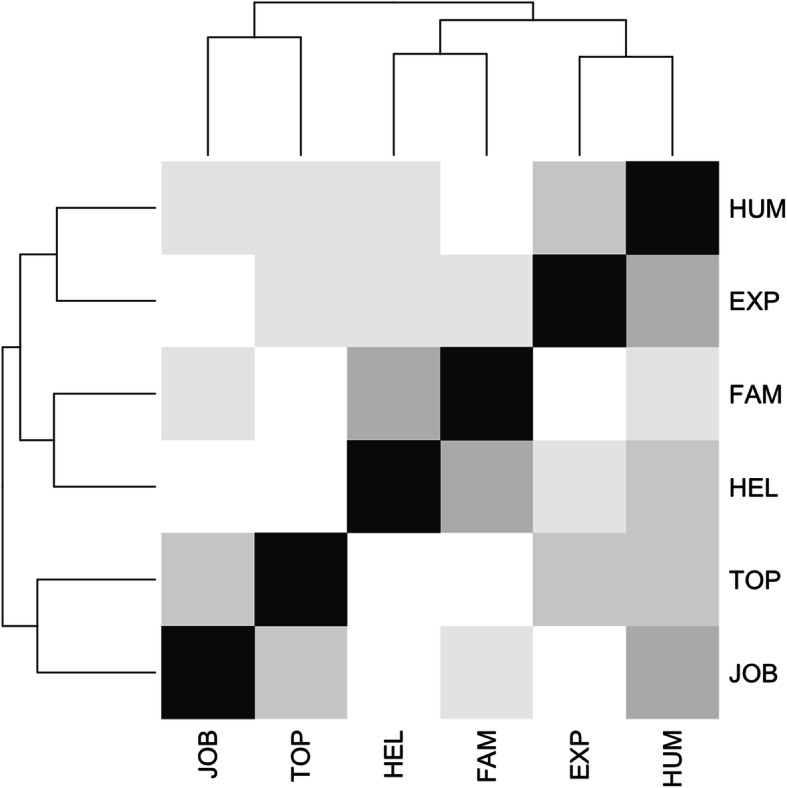
Associations among categories using the correlation matrix. The intensity of the colour grey indicates a stronger association; clusters are highlighted on the left and superior margins

The hierarchical cluster analysis suggested associations among different categories (see Fig. 1). The desire to help others (HEL) in association with the family’s influence (FAM) formed the first cluster of motivation. The second cluster was the association between healthcare-related experiences (EXP), such as voluntary work in healthcare settings or a family member’s hospitalisation, and the desire to enter into contact with other people (HUM). These two clusters were separated by the cluster formed by job opportunities (JOB) and personal interest in scientific topics (TOP).

This preliminary result seems to suggest a more complex motivational structure with respect to a dichotomous separation between internal and external motivational factors [27]. The internal motivational factors (EXP, HUM, HEL and FAM) can be subdivided into two clusters. The first cluster groups motivations oriented towards the individual (EXP and HUM), while the second groups the other two categories, which are more related to a prosocial attitude (HEL and FAM). The external dimension appears to be represented by the categories JOB and TOP.

As described in the method section, the PCA was used to conduct a multidimensional scaling with the aim to better understand these clusters of motivations. The very good compatibility between the outcomes of the PCA, with three factors and varimax rotation, and the previous hierarchical structure (Table 3) supported the validity of these findings.

**Table 3:** Component matrix for motivational categories

	RC1	RC2	RC3
EXP	−0.25	−0.54	**0.55**
HUM	0.07	0.14	**0.91**
HEL	**0.63**	−0.42	0.07
FAM	**0.68**	0.11	−0.10
JOB	−0.08	**0.88**	0.14
TOP	**−0.63**	0.01	−0.03

The highest absolute load for each motivational item is in bold

RCs are the three Rotated Components

Extraction Method: Principal Component Analysis

Rotation Method: Varimax with Kaiser Normalisation

The loads in Table 3 confirm the motivational structure in Fig. 1. In particular, the second rotated component, denominated RC2 in Table 3, appears connected to an external dimension, with job opportunities as the strongest motivational theme. The third component, denominated RC3, seems to correspond to internal motivations oriented to individual interests, with major loadings on the categories EXP and HUM related to personal experience and human personal contact, respectively. Finally, the RC1 component reflects the internal motivations with a prosocial orientation, and the categories HEL and FAM prevail – that is, categories oriented towards the needs of other people, with a negative correlation with TOP. Motivation and empathy scores are separately analysed in the subsequent part of this paper, after which correlations between motivation and the scores in the JSE questionnaire are examined.

Observing the frequencies of the different categories with respect to sex and age, some differences can be highlighted (see Table 2). In females’ motivations, the categories that refer to willingness to help and care, as well as healthcare related experiences, are recurrent (HEL, HUM, and EXP). In males, the highest reported motivations, other than the HEL category, are more oriented towards job security (JOB) and interest in scientific topics (TOP). This sex difference is statistically significant (*χ*^*2*^ = 34.22, *df* = 5; *p* < 0.01).

For the younger students (‘Young’ and ‘Mid’ classes in Table 2, age ≤ 21), the frequencies of the motivation categories are not different with respect to the total sample. Older students (‘Old’ class in Table 2, age >  21) were shown to give more importance to motivations related to job opportunities (JOB). These differences have a weaker statistical significance (*p* < 0.04).

### Empathy scores

Descriptive statistics regarding the JSE-HPS administered to the first-year students in the nursing degree course are reported in Table 4. The mean and standard deviation of the JSE-HPS empathy total scores and the statistical analysis are summarised. Considering the JSE scores reported in the literature [18, 32], the statistics that were obtained agree with the expected values and confirm a good external validity of the data collected in this sample.

**Table 4:** Descriptive statistics for JSE-HPS (N = 77 first-year nursing students)

Statistics	Total	Perspective taking	Compassionate care	Standing in patient’s shoes
Mean	111.88	57.6	44.69	9.60
Standard Deviation	11.90	7.35	5.39	2.66
25th percentile	104	53	41	8
50th percentile (median)	113	60	45	9
75th percentile	122	62	49	11
Possible Range	20–140	10–70	8–56	2–14
Actual Range	75–132	36–70	28–55	4–14
Cronbach’s alpha	0.79	0.65	0.58	0.56

Taking sex into account (Table 5), the mean of empathy scores was higher for female students (*M* = 114.90, *SD* = 10.20) than for males (*M* = 106.90, *SD* = 12.97). The non-parametric Kruskal-Wallis test confirmed a significant difference between the empathy total scores (*χ*^*2*^ = 6.73, *df* = 1; *p* < 0.01), and this difference is more significant if the subscale ‘Compassionate Care’ is considered (*χ*^*2*^ = 11.16).

**Table 5:** Comparisons of the total JSE-HPS scores for first-year nursing students with respect to different groups: sex, age, and motivational categories

Groups	n	M	SD	U test ^(a)^	*p*-value
*Sex*					
Male students	29	106.90	12.97	942.5	.005**
Female students	48	114.90	10.20		
All students	77	111.88	11.90		
*Age class*					
Young (≤ 19 years)	32	112.75	11.86	762	.332
Mid (> 19 and ≤ 21 years)	27	110.41	11.92	596	.199
Old (> 21 years)	18	112.56	12.39	568	.328
*Nursing motivation*					
Healthcare-related personal experiences (EXP)	14	112.86	15.38	499	.222
Human contact (HUM)	19	109.84	13.48	505	.293
The willingness to care for people (HEL)	57	112.95	9.80	614	.302
Family tradition (FAM)	4	113.00	11.25	186	.451
Job opportunities (JOB)	22	109.59	12.35	515.5	.156
Personal interest (TOP)	17	105.35	15.32	348.5	.024*

^(a)^ For the group comparison, the Mann-Whitney U non-parametric test was used, dividing the sample into two independent groups according to their membership status. The symbols (*) and (**) indicate levels of significance greater than 95% and 99%, respectively

Using an ordinary linear regression model, no significant correlation between the JSE scores and students’ age was found. The separate analysis of each of the three age classes (‘Young’, with age ≤ 19; ‘Mid’, with ages between 20 and 21; ‘Old’, with age >  21) confirmed differences among the groups. The study of the interaction between age and sex showed a significant effect for the differences in the total JSE scores only in the case of younger students, with young females’ total scores being higher than those of young males (*p* < 0.05). For the other two age classes, these differences were not statistically significant.

### Associations between the motivation for choosing nursing studies and JSE-HPS empathy scores

The data in Table 5 show that higher empathy scores are associated with internal motivation, particularly with the internal motivations with a prosocial orientation (HEL and FAM). The external motivational factors (JOB and TOP) are associated with mean scores below the average, but this difference is only significant for the TOP category.

The results of the PCA were used to obtain a more statistically significant analysis of the associations between motivation and empathy scores.

Starting from the components illustrated in Table 3, it is possible to obtain a personal score vector for each student in the sample, which summarises their position in the three-dimensional space defined by the three rotated components: RC1, RC2, and RC3. Consequently, all components can be regressed on the JSE-HPS scores. When the JSE scores are used, we obtain two interesting correlations: a positive correlation between the prosocial scores (RC1) and the JSE total scores, and a negative correlation between the external dimension scores (RC2) and the scores in the JSE-HPS subscale ‘Compassionate Care’ (see Table 6). No other analysis shows statistically significant results.

**Table 6:** Two regression models between the RC1 and RC2 dimensions for students’ motivation and JSE-HPS scores

	*Estimate*	*SE*	*t value*	*Pr (>|t|)*	
*Model: RC1 ~ JSE_TOT*					
(intercept)	−1.888	1.070	−1.765	0.0816	.
JSE_TOT	0.017	0.009	1.777	0.080	.
*Model: RC2 ~ JSE_CC*					
(intercept)	1.865	0.939	1.986	0.051	.
JSE_CC	−0.042	0.021	−2.000	0.049	*

JSE_TOT*:* JSE-HPS total score

JSE_CC*:* scores in the JSE-HPS subscale ‘Compassionate Care’

RC1 and RC2 the first two Rotated Component obtained in the PCA (Table 3)

## Discussion

### Association between empathy and reasons for enrolling in nursing

The primary aim of this study was to investigate the association between nursing students’ positive attitudes towards the value of empathy and their altruistic motivations for choosing to engage in nursing studies. The motivations of 77 first-year students for engaging in nursing studies were collected, and their empathy was measured with a broadly used and valid instrument, the Health Professions Students’ version of the JSE. We hypothesised a positive association between these two dimensions. First, the motivations to engage in nursing studies were examined through a thematic analysis. This analysis showed an interesting motivational structure, with the internal motivations appearing separately in two sub-dimensions. One sub-dimension was more related to individual interests, such as personal life experiences or interests in human contact. The other sub-dimension was more related to an altruistic stance, where an important role is played both by the desire to help other people and the family’s influence. The external extreme of the motivational scale includes motivations related to job opportunities. A large proportion of students indicated altruistic motivations for choosing nursing studies, corresponding to the literature on this topic [24–27].

The results confirmed the primary hypothesis that altruistic motivations for choosing nursing studies and students’ positive attitude towards the value of empathy in health professionals were significantly associated. Two relevant correlations emerged from the outcomes of this study. Internal motivations with a prosocial orientation were positively correlated with empathy scores. Moreover, when external motivations were considered, a negative correlation was found between these motivations and the JSE-HPS subscale that measures emotional engagement and compassionate care. The results obtained are interesting, as they confirm similar findings that were made for other typologies of health professionals. Previous studies investigated the association between empathy and person-oriented motives for enrolling in medical school [20, 22]. In one study, empathy was found to be weakly associated with person-centred motivations in recently admitted medical students [20]. Piumatti et al. showed that the internal motivational factors for studying medicine were associated with higher levels of empathy, while external motivational factors were associated with lower levels of empathy [22]. Another study reported that the empathy scores of first-year medical students were positively associated with their intention to pursue people-oriented specialties after graduation [21]. However, these findings do not correspond to those of the present study. Indeed, the targets of these studies were medical students rather than nursing students. Additionally, these studies utilised structured questionnaires and closed questions to measure motivational aspects. To the best of our knowledge, no prior studies have examined this association in the nursing sector.

The mixed methodology proposed in this study, in which the results of a qualitative analysis have been elaborated and associated with the scores of a quantitative survey, can offer a first proposal to reduce the gap between different research methods in nursing science. This can help the passage from the current ‘preparadigmatic state’ toward the adoption of more coherent and rigorous research methods [35].

### Age and sex differences in empathy scores and nursing motivations

The secondary aim of this study was to verify the association of nursing motivation and empathy scores with age and sex. The analysis of sex differences indicated that internal motivations were more present in the female students’ responses to the open question as opposed to male students, whose responses were more oriented towards external motivations. Furthermore, female students reported a higher mean score on the JSE; these results are consistent with those of other studies, in which women significantly reported higher empathy scores [20, 22, 31]. Different explanations have been given for sex differences in empathy scores, such as genetic predispositions and social learning.

When respondents’ age was analysed, no significant differences in motivation were observed. The only relevant difference concerned motivations related to job security, which was mainly indicated as a motivation for choosing nursing studies by students who were older than 21. These results are in accordance with the findings in previous research. In a prior study, male students’ self-reported motivations for choosing nursing studies were more oriented to job security [27]. Finally, the empathy scores were not significantly influenced by students’ age. The differences in the total JSE scores were shown only for younger students, with young females’ total scores being higher than those of young males.

### Limitations

This study has some limitations. The use of a qualitative analysis to detect motivations makes it difficult to generalise the results of this study to other subject samples. The use of a structured questionnaire could be more appropriate for generalising the results. The cross-sectional nature of the study prevents the evaluation of causal relationships between the observed variables. The inclusion of students from a single institution may also limit the generalizability of the findings of this study. Moreover, the relationship between empathy and motivation can evolve over time, and only a longitudinal study could provide a better understanding of this evolution. Another limitation was due to the fact that the students’ professor was also a researcher on this study; this may have biased the study results. Precautions were taken to reduce any students’ perceived pressure to participate in the study. Finally, an aspect to consider is social desirability. Owing to the nature of the construct, the process of measuring empathy is often deeply affected by a social desirability bias and acquiescence. Therefore, future research should use a different typology of measurements, such as implicit measures of empathy, or measurements of patients’ perceptions of health professionals’ empathy.

## Conclusions

The main finding in this study shows that first-year nursing students’ high empathy scores are positively associated to altruistic and prosocial motivations for choosing nursing studies. This result can help to guide further research to better understand the empathy construct, particularly regarding the correlation of specific empathy components with motivational dimensions, such as willingness to feel useful and to affect the health of others. Moreover, considering that empathy is a modifiable and trainable dimension, knowing the level of nursing students’ empathy is important for educators, as it allows them to implement training paths to slow the decrease in empathy or to enhance students’ relational attitudes and skills, which are central aspects for this profession. Furthermore, it is crucial to promote specific interventions and activities to support or reinforce the positive motivational aspects that students already possess and that are essential for the nursing profession.

## Data Availability

The datasets analysed during the current study are available from the corresponding author on reasonable request.

## Abbreviations

JSE: Jefferson Scale of Empathy
JSE-HPS: Jefferson Scale of Empathy - Health Professions Students
EFA: Explorative Factorial Analysis
